# Oral lichen planus clinical characteristics in Italian patients: a retrospective analysis

**DOI:** 10.1186/s13005-016-0115-z

**Published:** 2016-04-26

**Authors:** Dorina Lauritano, Mariantonietta Arrica, Alberta Lucchese, Marina Valente, Giuseppe Pannone, Carlo Lajolo, Rossella Ninivaggi, Massimo Petruzzi

**Affiliations:** Department of Translational Medicine and Surgery Neuroscience Centre of Milan, University of Milano Bicocca, Monza, Italy; Dental School of University of Sassari, Sassari, Italy; Dental Clinic of Second Universty of Naples (SUN), Naples, Italy; Section of Pathology of University of Foggia, Foggia, Italy; School of Dentistry, Chatolic University, Rome, Italy; Interdisciplinary Department of Medicine (DIM) - Section of Dentistry, University “Aldo Moro” of Bari, Clinica odontoiatrica del Policlinico di Bari, Piazza Giulio Cesare 11, 70124 Bari, Italy

**Keywords:** Oral lichen planus, Retrospective study, Autoimmune diseases

## Abstract

**Background:**

Oral lichen Planus (OLP) is a chronic inflammatory disease involving skin and mucous membranes. Its etiology is still uncertain whilst an autoimmune mechanism is known to be implicated. OLP is commonly considered a geriatric disease and gender differences in prevalence are clear, whereby females are generally more frequently affected than males more often during the 5th and 6th decades of life. Lesions are symmetrical and bilateral and the buccal mucosa is frequently involved. The risk of malignant transformation is extremely low.

This study aims to describe both the clinical characteristics and the prevalence of OLP among a group of patients from Southern Italy. The results of the present study were compared to analogous retrospective studies.

**Methods:**

Eighty-seven (31 man and 56 woman) cases of OLP were retrospectively reviewed and demographic and clinical data were collected. Data about OLP as clinical forms, oral and extraoral sites involved and Visual Analogue Scale were also recorded.

**Results:**

The average age of OLP onset was 59.2 years. The most common clinical presentation was the hyperkeratosic type. Symptomatic OLP was noted in 26.8 % of the patiens. The most frequently affected oral sites were buccal mucosa, tongue, gums. The most frequently associated systemic diseases were diabetes, hypertension, C hepatitis and thyroiditis. Only one patient developed a malignant transformation (1.2 %).

**Conclusions:**

Previous retrospective studies report data partially comparable with our results. Different geographic area, number of enrolled patients and OLP classification criteria may justify the observed differences.

## Background

Lichen planus is a quite common chronic inflammatory disease of the skin and mucous membranes, in which the immunological system is believed to play a leading role [1]. It affects all racial groups whit an estimated incidence of 0.5 %–1.5 % [2, 3]. The etiology of the oral lichen planus (OLP) remains still unclear, but probably an unknown antigen alters the basal keratinocytes of the oral mucosa making them a target of the cell immune response. CD4^+^ T and CD8^+^ T lymphocytes, producing cytokines, interleukin-2 and tumor necrosis factor, induce a chronic inflammatory response and the keratinocytes apoptosis [4]. Variable degrees of ortho or parakeratosis, acanthosis, hypergranulosis and vacuolar degeneration of basal keratinocytes are a consequence of a maturation delay. Moreover, the inflammatory origin of the disease is proved by the marked layered lymphocytic infiltrate (“band-like”) immediately underlying the epithelium [5]. The papule is the elementary lesion that characterize the OLP: their confluence can generate white reticular striae (Wickham striae) or plaques [3]. Papular, reticular and plaque-like OLP are considered as hyperkeratosic or “white form”, usually no symptomatic. On the contrary, atrophic, erythematosus, erosive and bullous variants of OLP are symptomatic and distinguished as “red form” [6]. The OLP lesions are usually bilaterally/symmetrically localized and the most common involved sites are the buccal mucosa, tongue, gingiva, lips, floor of mouth, palate. Oral lesions may appear weeks or months before the appearance of cutaneous, oesophageal or genital lesions [7, 8].

OLP diagnosis should be done by clinical and histological examination: Van der Maij and Van der Wall postulated a list of clinical and histopatological features that could help in the diagnosis formulation [9]. In particular, the clinical features include bilateral oral lesions and reticular lesions; in case of absence of reticular lesions they suggest to use the term “clinically compatible with OLP”. The proposed histopathological features include the presence of liquefaction and degeneration of basal cells and band-like infiltrate of lymphocytes and absence of epithelial dysplasia [9]. The absence of epithelial dysplasia is mandatory to distinguish OLP form lichenoid dysplasia, in fact lichenoid dysplasia may share whit OLP the clinical appearance but not the risk of a neoplastic derailment [10]. The rate of malignant transformation of OLP seems to be lowest than lichenoid dysplasia (1.09 vs. 3.20) but an accurate and prolonged follow-up period is also necessary for OLP patients [11].

Although a recent metanalytic review pointed out *“the insufficient evidence to support the effectiveness of any specific treatment*”, a clinical management of OLP patients is often required, above all in symptomatic patients [12]. The treatment of OLP is no curative, in fact its efficacy is related to the symptoms control and ulcers management (especially in erosive OLP). The asymptomatic forms do not warrant management with drugs; they are only supposed to be periodically followed up by clinicians. On the contrary, symptomatic OLP should be treated whit high topical potency corticosteroids, systemic prednisone or immunosuppressive agents like cyclosporine, tacrolimus and thalidomide [13].

The present study aims to describe a cohort of patients from the south of Italy affected by OLP and aims to compare our data with the data from similar case studies reported in Literature.

## Methods

This retrospective study analyzed all consecutive patients affected by OLP referred to the Dental Clinic of University of Bari between January 2011 and June 2014.

Demographic and clinical data from recent and remote anamnesis including gender, age, systemic disorders and related medications were collected. Data about OLP clinical forms, oral and extraoral sites involved and a quantification of the initial symptoms obtained through the Visual Analogical Scale (VAS) were also recorded. The VAS is a continuous scale for recording pain intensity; it ranges from 0 (no pain) to 10 (worst pain) [14].

Statistical differences were calculated in the distribution of different types of OLP in men and women using chi-square test while VAS differences, OLP clinical form distribution in sex and age, were calculated with the ANOVA test.

Results were considered statistically significant if *p* < 0.05.

All patients were diagnosed as OLP applying the Van der Meij and Van der Wall criteria (symmetrical distribution of lesions and grid-shaped lesions) and consequential histological examination [9].

Clobetasol propionate ointment in orabase, systemic corticosteroids and topical calcineurin inhibitors were used alone or in combination to treat OLP signs and symptoms.

This study was approved by the local ethical committee (study n. 4743 – Ethical Committee of Policlinico di Bari) and conducted according to the Declaration of Helsinki. All patients signed a informed consent for the treatment of their clinical data and publication.

## Results

Eighty-seven patients met the Van der Meij and Van der Wall criteria [9]. In the Tables 1 and 2 are reported the demographical data and comorbidities in OLP patients. The male:female ratio was 1:1.8 (31 men and 56 women). The mean age of the patients was 63.9 years (range 27–93). The average age of onset of the disease was 59.2 years. The mean age at presentation was 56.5 for women and 61.8 for men (*p* value = 0.03). Sixty-seven patients (77 %) presented one or more concomitant diseases, while the remaining 20 patients (23 %) were in good health and did not take any drug. The more frequent OLP associated pathologies included hypertension (44.8 %, 39 subjects), diabetes (24.1 %, 21 subjects), C hepatitis (active disease and/or patients who received treatment −18.4 %, 16 subjects), thyroiditis (15 %, 13 subjects), psychiatric disorders as depression and anxiety, (10.3 %, 9 subjects), other systemic diseases as heart disease, prostatitis, hypercholesterolemia (31 %, 27 subjects). Six-teen patients were both affected by diabetes and hypertension (Grinspan syndrome) [8]. Four patients (5 %) also presented cutaneous lesions related to lichen planus. Antihypertensive and antidiabetic medications were the most used medications in our cohort of patients, taken alone or in combination. Only 20 OLP patients did not present associated diseases and did not take any drugs or medications.

**Table 1:** Demographical characteristics and associated diseases in analyzed cohort of OLP patients

	Patients (n)	Mean age (years)	Mean age at the OLP onset (years)	OLP associated disease (n.patients-%)
Men	31	63.1	61.8	OLP Associated disease: 67^a^–77 % - Hypertension 39–44.8 % - Diabetes- 21–24.1 % - B&C hepatitis 16–18.4 % - Thyroiditis 13–15 % - Depression & anxiety 9–10.3 % No associated disease 20–23 %
Women	56	64.4	56.5	
Total	87	63.9	59.15	

^a^48 patients (55 %) were affected by more than one disease

**Table 2:** Demographical characteristics and associated diseases in analyzed cohort of OLP patients

Medications taken alone or in combination (n.patients-%)					
Antihypertensive 39–44.8 %	Antidiabetic agents 21–24.1 %	Interferon 14–16 %	Ribavirin 12–14 %	Ansyolitics 12–14 %	Antidepressive 10–11.5 %
Hormones 15–17.2 %	Antiaggregants 5–6 %	Cardioaspirin 25–28.7 %	Other medications 6–7 %	No medications 20–23 %	

**Fig. 1:**
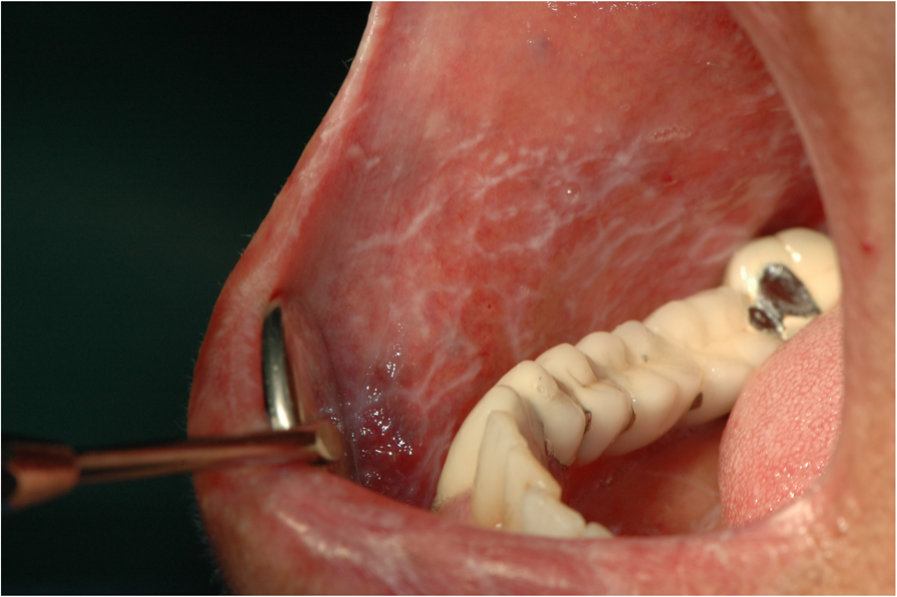
Classical clinical aspect of a reticular oral lichen planus

**Fig. 2:**
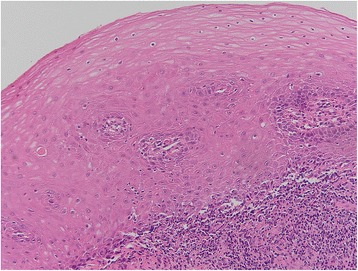
Hyperkeratotic OLP showing hyperkeratosis, bandlike monomorphic lymphocytic infiltrate, liquefactive degeneration of basal epithelial cells creating small Max Joseph spaces (*H&E, original magnification x10*)

**Fig. 3:**
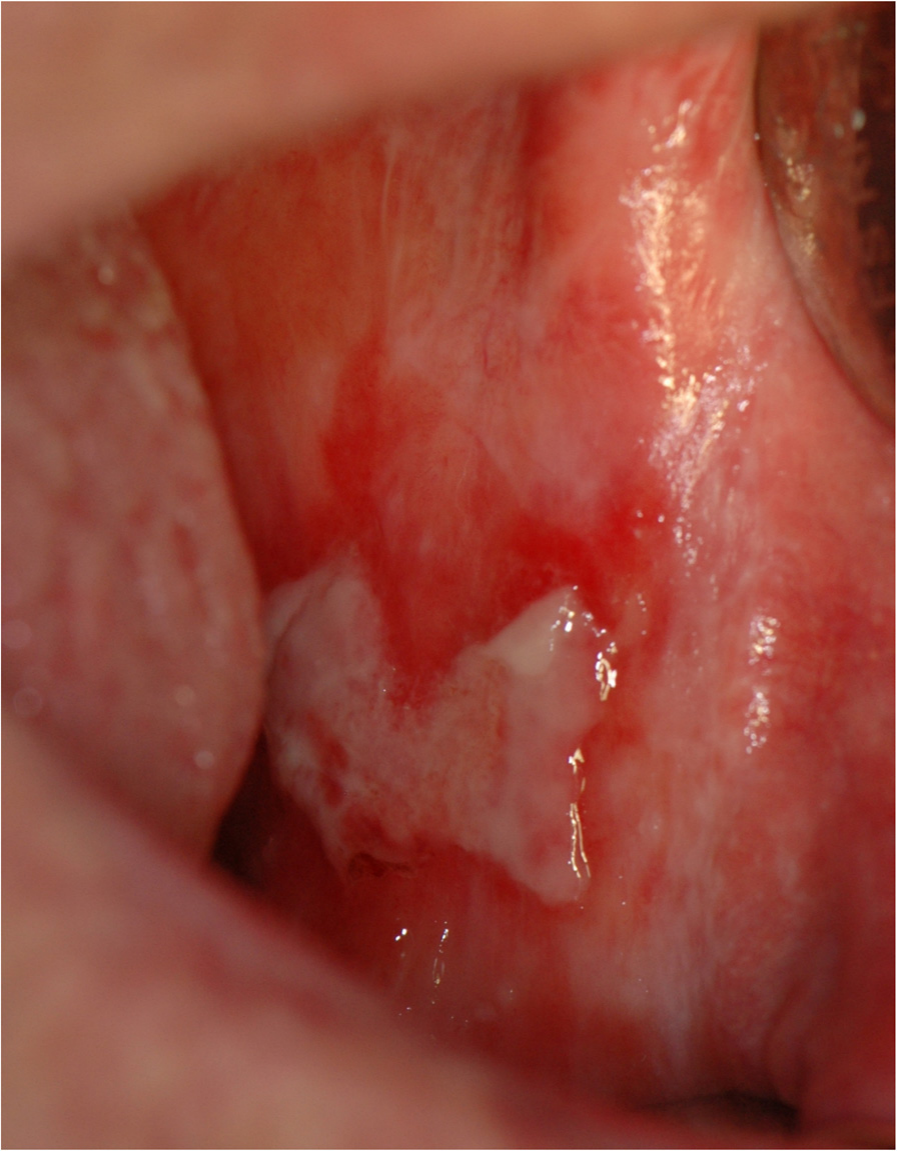
Erosive OLP involving the buccal mucosa. A pseudomembrane cover the erosive area

**Fig. 4:**
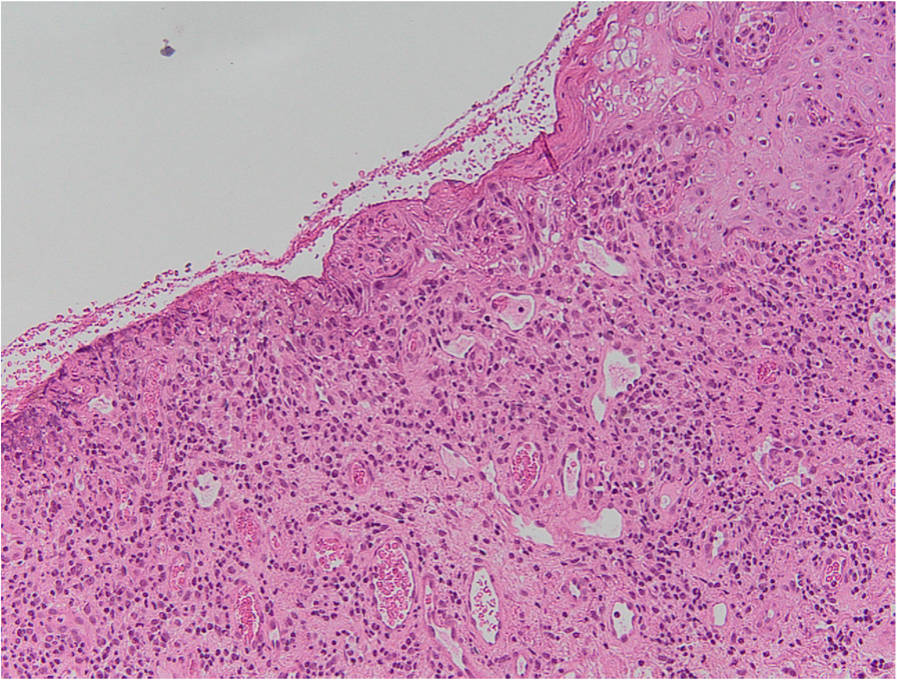
Erosive area in OLP (*H&E, original magnification x10*)

In Tables 3 are shown the data related to the OLP in the analyzed sample. Hyperkeratosic OLP (white forms- Figs. 1 and 2) was observed in 53 patients (58.5 %) while red OLP (Figs. 3 and 4) was noted in 34 patients (39 %). Red forms of OLP were diagnosed in 44.7 % of the women while the remaining 55.3 % reported white forms. Also in men, the most frequent clinical OLP form was the white one (67.7 %) while only one-third (32.3 %) of them reported the red forms. Older patients tends to develop red forms of OLP (mean age at the onset 59.7 years) while the white ones appears in younger patients (mean age at the onset 57.8 years) but no statistical difference was noted (*p* > 0.05). Mean age of patients at the onset of OLP white forms was 61.1 and 54.6 years for male and female respectively. Mean age of patients at the onset of OLP red forms was 54.1 and 65.3 years for male and female respectively. The mean duration of OLP lesions was 65 months with no significant differences (*p* > 0.05) between male (62.7) and female (67.4). A total of 46 patients (52.9 %) were symptomatic, 34 (73.9 % of the symptomatic patients) of these subjects showed an atrophic erosive OLP, while the remaining 8 symptomatic patients suffered from hyperkeratosic OLP. VAS was higher in male than in female (3.4 vs. 3.3) with a mean VAS of 3.3 but ANOVA did not show any statistical difference (*p* > 0.05). OLP lesions were diagnosed in different mucosal areas, coexisting simultaneously. In particular, the buccal mucosa was the most frequent OLP involved oral site (in 56 patients - 64.4 %) while the lateral border and dorsal region of the tongue were involved in 31 patients (35.6 %). OLP lesions on gingival mucosa were noted in 20 patients (23 %) while the involvement of hard palate (7 patients, 8 %) fornix (6 patients, 6.9 %), lip mucosa (6 patients, 6.9 %) and soft palate (3 patients, 3.5 %) was observed less frequently.

**Table 3:** Clinical characteristics of OLP lesions (signs, symptoms and malignant transformation)

	Clinical Type of OLP (n. patients)	Age at the OLP onset (years)	Duration of OLP (months)	VAS	Malignant Transformation	Involved mucosal sites (n. patients-%)
Men			62.7	3.4 ± 2.7	1 case	Buccal (56–44.7 %) Tongue (31–35.6 %) Gums (20–23 %) Hard palate (7–8 %) Fornix (6–6.9 %) Lip (6–6.9 %) Soft palate (3–3.5 %)
White forms	21	61.1				
Red forms	10	54.1				
Women			67.4	3.3 ± 3.0	0	
White forms	32	54.6				
Red forms	24	65.3				
Total	53 white forms 34 red forms	57.8 white forms 59.7 red forms	65.0	3.3 ± 2.9	1 case

**Table 4:** Comparison with existing knowledge

Study	Country	Patients number	F:M	Mean Age	The 3 most affected oral sites	Associated diseases	Most frequent OLP clinical form	Rate of malignant transformation
Salem G. (1989) [28]	Saudi Arabia	4277	n.r.	49	buccal mucosa, gingiva, tongue	no association	atrophic- erosive	0.02 %
Bagan-Sebastian J.V. (1992) [29]	Spain	205	4:1	52.6	buccal mucosa, gingiva, tongue	diabetes	atrophic- erosive	n.r.
Gorsky M. et al. (1996) [30]	Israel	157	1.5:1	52.5	buccal mucosa, gingiva, tongue	no association	reticular	1.3 %
Rossi L. et al. (2000) [31]	Italy	100	1.32:1	58.6	buccal mucosa, gingiva, tongue	C & B hepatitis, diabetes	atrophic- erosive	n.r.
Eisen D. et al. (2002) [32]	USA	723	3:1	52	buccal mucosa, gingiva, tongue	C hepatitis	reticular	0.8 %
Torrente-Castell et al. (2010) [33]	Spain	65	1.56:1	59	buccal mucosa, gingiva, tongue	no association	hyperkeratotic	3.1 %
Xue J.L. (2005) [34]	China	674	1.93:1	50.4	buccal mucosa, lip, tongue	no association	reticular	0.6 %
Ingafou M. (2006) [35]	England	690	1.75:1	52	buccal mucosa, gingiva, tongue	no association	reticular	1.9 %
Pakfetrat A. (2009) [36]	Iran	420	1.85:1	41.6	buccal mucosa, gingiva, tongue	no association	reticular	0.7 %
Bermejo-Fenoll A. (2010) [37]	Spain	550	3.29:1	56.4	buccal mucosa, gingiva, tongue	hypertension, rheumatic diseases gastrointestinal disorders, anxiety & depression	reticular and papular	0.9 %
Shen Z.Y. (2012) [38]	China	518	2.13:1	46.3	buccal mucosa, gingiva, tongue	no association	reticular	1 %
Gümrü B. (2013) [39]	Turkey	370	2.36:1	49.8	buccal mucosa, gingiva, tongue	hypertension, diabetes, anxiety & depression	atrophic- erosive	0.3 %
Present study	Italy	87	1.8:1	58.8	buccal mucosa, gingiva, tongue	hypertension, Diabetes, thyroiditis, C hepatitis	hyperkeratotic	1.15 %

*n.r* not reported

Only one (1.2 %) male patient (no smoker) developed a squamous cell carcinoma on his left cheek after 5 years from OLP diagnosis. The neoplasm was completely removed (excisional biopsy) and no recurrence was noted after 5 years. None of the remaining 86 patients developed a squamous cell carcinoma.

## Discussion

The present study confirms that OLP can be considered a geriatric pathology with a light preponderance among female in 5th and 6th decade. In our cohort of patients, the most frequent associated comorbidity was the hypertension: as often reported in literature, this association can easily explain the nature of oral lesions related to the assumption of antihypertensive drugs [15, 16]. On the other hand, the concomitant presence of these diseases could be completely accidental and compatible with the mean age of the patients. Diabetes has been also supposed to play a role in the OLP pathogenesis in fact patients with OLP and diabetes also seem to present more aggressive (atrophic-erosive) oral lesions [17]. This data may suggest the importance of vascular and neurological factors in the OLP pathogenesis. Grinspan syndrome is characterized by three simultaneous symptoms: hypertension, diabetes mellitus and erosive OLP but Lamey et al. [18] supposed that oral lesions were induced by antihypertensive drugs, frequently taken by diabetic patients.

Even if it has been analyzed in many studies, the cause of the association between the OLP and thyroid's pathologies is still unclear. Two studies showed a very high frequency of anti-nuclear antibodies (ANA), gastric parietal cell antibodies (GPCA), autoantibodies against thyroglobulin (TGA), microsomal anti fraction autoantibodies (TMA) in the serum of the patients affected by OLP [19, 20]. Other authors suggest that the circulating thyroid antibodies, in patients affected by Hashimoto’s thyroiditis, may contribute to trigger specific autoimmune response, to the skin and/or to the oral mucosa, moreover patients suffered from thyroid diseases represents a subset of OLP patients [21, 22].

The prevalence of the C hepatitis in our sample was considerably high (18.4 %): association between OLP and HCV still remain a debatable issue but a recent meta-analysis shows an estimate risk 6 times greater of HCV hepatitis in OLP patients [23]. This data supports an etiopathological association between C hepatitis and OLP in some geographical areas. The immunological imbalance due to virus activity may trigger the OLP pathogenesis, in predisposed patients [24]. According to Carrozzo et al., HLA-DR6 may be responsible for geographical peculiarities of the HCV-OLP association [25].

World Health Organization (WHO) classify OLP as a potentially malignant disorder although the annual malignant transformation is set at a percentage of less than 0.5 % [26, 27]. There are no known predictive factors (clinical and histopathological) of malignant transformation of OLP and to date it is not possible to prevent future cancer development from OLP lesions [27].

Oral examination at least once/twice a year still remain the most effective preventive measure in order to early detect the neoplastic derailment of OLP.

Comparing our results whit previous retrospective studies on OLP (Table 4), appears that OLP has a light predilection for women like as the majority of autoimmune diseases [28–39]. The diagnosis is usually made at the 5th decade of life, according to clinical and histopathological criteria. In all analyzed studies, the OLP lesions were bilateral and symmetrical, involving more than one site at the same time: buccal mucosa, gingiva and tongue are the three oral mucosal sites more frequently involved by OLP lesions. Malignant transformation of OLP lesions has been detected in all the analyzed studies in Table 4, exception for two of them [29–31]. The average prevalence of malignant transformation in the OLP lesions was 0.95 %, anyways the onset of oral cancer in a cohort of patients affected by OLP is comparable to the onset of cancer in the general population. According to WHO data OLP is considered a lesions with a low potential for malignant transformation. Lesions susceptible to malignant transformation belong to the red forms.

## Conclusions

The data about the associations between OLP and systemic disease are diversified. In the majority of the studies there is no significant relevance, on the contrary in this study the percentage of patients affected by disease as diabetes, hypertension, hepatitis C and thyroiditis turns out to be much higher than the prevalence of this disease in the general population. The geriatric age may contribute to the presence of co-morbidities in OLP patients and probably also their polypharmacolgical treatment play an important role in OLP pathogenesis and chronicization.

## References

[1] Baccaglini L, Thongprasom K, Carrozzo M, Bigby M (2013). Urban legends series: lichen planus. Oral Dis.

[2] McCartan BE, Healy CM (2008). the reported prevalence of oral lichen planus: a rewiew and critique. J Oral Pathol Med.

[3] Di Stasio D, Guida A, Salerno C, Contaldo M, Esposito V, Laino L, Serpico R, Lucchese A (2014). Oral lichen planus: a narrative review. Front Biosci (Elite Ed).

[4] Payeras MR, Cherubini K, Figueiredo MA, Salum FG (2013). Oral lichen planus: focus on etiopathogenesis. Arch Oral Biol.

[5] Gupta S, Jawanda MK (2015). Oral Lichen Planus: An Update on Etiology, Pathogenesis, Clinical Presentation,Diagnosis and Management. Indian J Dermatol.

[6] Carbone M, Arduino PG, Carrozzo M, Gandolfo S, Argiolas MR, Bertolusso G (2009). Course of oral lichen planus: a retrospective study of 808 northern Italian patients. Oral Dis.

[7] Petruzzi M, De Benedittis M, Pastore L, Grassi FR, Serpico R (2005). Peno-gingival lichen planus. J Periodontol.

[8] Petruzzi M, De Benedittis M, Carriero C, Giardina C, Parisi G, Serpico R (2005). Oro-vaginal-vulvar lichen planus: report of two new cases. Maturitas.

[9] Van der Meij EH, van der Waal I (2003). Lack of clinicopathologic correlation in the diagnosis of oral lichen planus based on the presently available diagnostic criteria and suggestions for modifications. J Oral Pathol Med.

[10] Czerninski R, Zeituni S, Maly A, Basile J (2015). Clinical characteristics of lichen and dysplasia vs lichen planus cases and dysplasia cases. Oral Dis.

[11] Fitzpatrick SG, Hirsch SA, Gordon SC (2014). The malignant transformation of oral lichen planus and oral lichenoid lesions: a systematic review. J Am Dent Assoc.

[12] Keenan AV, Ferraiolo D (2011). Insufficient evidence for effectiveness of any treatment for oral lichen planus. Evid Based Dent.

[13] Lodi G, Scully C, Carrozzo M, Griffiths M, Sugerman PB, Thongprasom K (2005). Current controversies in oral lichen planus: report of an international consensus meeting. Part 2. Clinical management and malignant transformation. Oral Surg Oral Med Oral Pathol Oral Radiol Endod.

[14] Burckhardt CS, Jones KD (2003). Adult measures of pain: The McGill Pain Questionnaire (MPQ), Rheumatoid Arthritis Pain Scale (RAPS), Short-Form McGill Pain Questionnaire (SF-MPQ), Verbal Descriptive Scale (VDS), Visual Analog Scale (VAS), and West Haven-Yale Multidisciplinary Pain Inventory (WHYMPI). Arthritis Rheum.

[15] Ben Salem C, Chenguel L, Ghariani N, Denguezli M, Hmouda H, Bouraoui K (2008). Captopril-induced lichen planus pemphigoides. Pharmacoepidemiol Drug Saf.

[16] Kaomongkolgit R (2010). Oral lichenoid drug reaction associated with antihypertensive and hypoglycemic drugs. J Drugs Dermatol.

[17] Torrente-Castells E, Figueiredo R, Berini-Aytés L, Gay-Escoda C (2010). Clinical features of oral lichen planus. A retrospective study of 65 cases. Med Oral Patol Oral Cir Bucal.

[18] Lamey PJ, Gibson J, Barclay SC, Miller S (1990). Grinspan’s syndrome: a drug-induced phenomenon?. Oral Surg Oral Med Oral Pathol.

[19] Chang JY, Chiang CP, Hsiao CK, Sun A (2009). Significantly higher frequencies of presence of serum autoantibodies in Chinese patients with oral lichen planus. J Oral Pathol Med.

[20] López-Jornet P, Parra-Perez F, Pons-Fuster A (2014). Association of autoimmune diseases with oral lichen planus: a cross-sectional, clinical study. J Eur Acad Dermatol Venereol.

[21] Lo Muzio L, Santarelli A, Campisi G, Lacaita M, Favia G (2013). Possible link between Hashimoto’s thyroiditis and oral lichen planus: a novel association found. Clin Oral Investig.

[22] Robledo-Sierra J, Landin-Wilhelmsen K, Nyström HF, Mattsson U, Jontell M (2015). Clinical characteristics of patients with concomitant oral lichen planus and thyroid disease. Oral Surg Oral Med Oral Pathol Oral Radiol.

[23] Hepatitis c virus infections in oral lichen planus: a systematic review and meta-analysis. Alaizari NA, Al-Maweri SA, Al-Shamiri HM, Tarakji B, Shugaa-Addin B. Aust Dent J. 2015 Oct 17. [Epub ahead of print] 10.1111/adj.12382 26475515

[24] Petruzzi M, De Benedittis M, Loria MP, Dambra P, D’Oronzio L, Capuzzimati C (2004). Immune response in patients with oral lichen planus and HCV infection. Int J Immunopathol Pharmacol.

[25] Carrozzo M, Brancatello F, Dametto E, Arduino P, Pentenero M, Rendine S (2005). Hepatitis C virus-associated oral lichen planus: is the geographical heterogeneity related to HLA-DR6?. J Oral Pathol Med.

[26] WHO Collaborating Centre For Oral Precancerous Lesions (1978). Definition of leukoplakia and related lesions: an aid to studies on oral precancer. Oral Surg Oral Med Oral Pathol.

[27] van der Waal I (2014). Oral potentially malignant disorders: is malignant transformation predictable and preventable?. Med Oral Patol Oral Cir Bucal.

[28] Salem G (1989). Oral lichen planus among 4277 patients from Gizan, Saudi Arabia. Community Dent Oral Epidemiol.

[29] Bagan-Sebastian JV, Milian-Masanet MA, Penarrocha-Diago M, Jimenez Y (1992). A clinical study of 205 patients with oral lichen planus. J Oral Maxillofac Surg.

[30] Gorsky M, Raviv M, Moskona D, Laufer M, Bodner L (1996). Clinical characteristics and treatment of patients with oral lichen planus in Israel. Oral Surg Oral Med Oral Pathol Oral Radiol Endod.

[31] Rossi L, Colasanto S (2000). Clinical considerations and statistical analysis on 100 patients with oral lichen planus. Minerva Stomatol.

[32] Eisen D (2002). The clinical features, malignant potential, and systemic associations of oral lichen planus: a study of 723 patients. J Am Acad Dermatol.

[33] Petrou-Amerikanou C, Markopoulos AK, Belazi M, Karamitsos D, Papanayotou P. Prevalence of oral lichen planus in diabetes mellitus according to the type of diabetes. Oral Dis. 1998;4(1):37–40. 10.1111/j.1601-0825.1998.tb00253.x 9655043

[34] Xue JL, Fan MW, Wang SZ, Chen XM, Li Y, Wang LA (2005). Clinical study of 674 patients with oral lichen planus in China. J Oral Pathol Med.

[35] Ingafou M, Leao JC, Porter SR, Scully C (2006). Oral lichen planus: a retrospective study of 690 British patients. Oral Dis.

[36] Pakfetrat A, Javadzadeh-Bolouri A, Basir-Shabestari S, Falaki F (2009). Oral Lichen Planus: a retrospective study of 420 Iranian patients. Med Oral Patol Oral Cir Bucal.

[37] Bermejo-Fenoll A, Sánchez-Siles M, López-Jornet P, Camacho-Alonso F, Salazar-Sánchez N (2010). A retrospective clinicopathological study of 550 patients with oral lichen planus in south-eastern Spain. J Oral Pathol Med.

[38] Shen ZY, Liu W, Zhu LK, Feng JQ, Tang GY, Zhou ZT (2012). A retrospective clinicopathological study on oral lichen planus and malignant transformation: analysis of 518 cases. Med Oral Patol Oral Cir Bucal.

[39] Gümrü B (2013). A retrospective study of 370 patients with oral lichen planus in Turkey. Med Oral Patol Oral Cir Bucal.

